# Dosimetric parameters and safety analysis of 3D-printing non-coplanar template-assisted interstitial brachytherapy for non-centrally recurrent cervical cancer

**DOI:** 10.3389/fonc.2023.1174470

**Published:** 2023-10-26

**Authors:** Cong Wang, Yan Cheng, Yadong Song, Jia Lei, Yiqian Li, Xia Li, Huirong Shi

**Affiliations:** ^1^ Department of Gynecological Radiation Oncology, The First Affiliated Hospital of Zhengzhou University, Zhengzhou, China; ^2^ Department of Gynecology, The First Affiliated Hospital of Zhengzhou University, Zhengzhou, China

**Keywords:** non-centrally recurrent cervical cancer, interstitial brachytherapy, 3D-printing, non-coplanar template, dosimetry

## Abstract

**Introduction:**

The prognosis of patients with non-central recurrent cervical cancer (NRCC) remains poor, and treatment options are limited. We aimed to explore the accuracy and safety of the 3D-printed non-coplanar template (3D-PNCT)-assisted ^192^Ir interstitial brachytherapy (ISBT) in the treatment of NRCC.

**Material and methods:**

A total of 36 patients with NRCC who received 3D-PNCT-guided ^192^Ir ISBT in the First Affiliated Hospital of Zhengzhou University from January 2021 to July 2022 were included in this study. There were 36 3D-PNCTs that were designed and printed. The prescribed dose was 30–36 Gy, divided into five to six times, once a week. To evaluate whether the actual parameters were consistent with the preoperative design, the dosimetric parameters of pre- and postoperative treatment plans were compared, including dose of 90% high-risk clinical target volume (HR-CTV D90), volume percentage of 100% and 150% prescribed dose V_100%_ and V_150_%, homogeneity index (HI), conformal index (CI), external index (EI), and dose received by 2 cm^3^ (D2cm^3^) of the rectum, colon, bladder, and ileum. The safety parameters including occurrence of bleeding, infection, pain, radiation enteritis, and radiation cystitis within 3 months after operation were recorded.

**Results:**

All patients successfully completed the treatment and achieved the goals of the preoperative plan. There was no significant difference in the accuracy (HRCTVD90, V_100%_, EI, CI, and HI) and safety (D2cm^3^ of rectum, colon, bladder, and ileum) parameters of the postoperative plan compared with the preoperative plan (all *p*>0.05). Major side effects included bleeding at the puncture site (13.9%), postoperative pain (8.3%), acute radiation cystitis (13.9%), and radiation enteritis (19.4%). There were no serious perioperative complications and no grade 3–4 acute radiotherapy side effects.

**Conclusion:**

3D-PNCT-assisted ^192^Ir ISBT can be accurately and safely applied in the treatment of patients with NRCC.

## 1 Introduction

Cervical cancer is one of the most common cancers that threaten women’s health worldwide (1). Around 29%–38% of patients with cervical cancer relapse following treatment, and around 75% of recurrent lesions are confined to the pelvic cavity. The 5-year survival rate of patients with central recurrence is 63%, whereas it is less than 10% for those with non-central recurrence (2). Treatment is especially challenging for patients who develop non-central recurrent diseases. Non-centrally recurrent cervical cancer generally refers to cervical cancer with recurrent lesions eccentric and adjacent to vital vessels, nerves, and organs located deep in the abdomen and pelvis (3, 4).

Non-centrally recurrent cervical cancer requires a comprehensive treatment plan. However, since these patients frequently undergo multiple and multiline treatments, the dose of external radiotherapy is limited. Therefore, the treatment carries a high risk and has low efficacy (2). Addressing this issue has consistently presented a significant clinical challenge that needs an immediate solution (5). High-dose-rate interstitial brachytherapy (ISBT) is an important method for the treating non-central recurrent cervical cancer. However, the application of ISBT in non-central recurrent cervical cancer is limited, mainly due to the lack of reliable guidance methods. Since the spatial distribution (including distance, depth, and angle) of the needles is difficult to control, the treatment is highly dependent on the personal clinical experience of the doctor, and the treatment effect is biased. Therefore, placing the applicator accurately, safely, and efficiently is the biggest challenge for clinicians when applying ISBT to treat non-central recurrent cervical cancer.

With the widespread application of 3D printing technology in clinical practice (6–10), the ingenious combination of 3D printing technology and ISBT is expected to solve these challenges. The customized 3D printing non-coplanar template (3D-PNCT) template contains the simulated needle tract information and the characteristic information of the body surface and internal important blood vessels, nerves, organs, and bones of the patient. It has the ability to position, orient, and avoid critical structures, thereby meeting the clinical requirements for high precision, high safety, easy operation, and good repeatability of the operation.

Therefore, we investigate the dosimetric changes and safety concerns associated with 3D-PNCT- assisted ISBT for treating non-centrally recurrent cervical cancer patients. The study aimed to assess the accuracy and safety of this advanced technology, which will contribute to a better understanding of its clinical applications. By providing evidence of its value and potential for widespread adoption, it will enhance the utilization and efficacy of 3D-PNCT-assisted ISBT in treating non-centrally recurrent cervical cancer.

## 2 Materials and methods

### 2.1 Study design and population

This retrospective study included 36 non-centrally recurrent cervical cancer patients treated with 3D-PNCT-assisted ISBT between January 2021 and July 2022, as is shown in **Table 1**. The inclusion criteria were as follows (1): age 18–70 years (2); performance status score of 0–1 (3); pathologically diagnosed squamous cell carcinoma or adenosquamous carcinoma; (4) recurrent lesions located deep in the abdomen and pelvis, more than 10 cm from the body surface (for superficial lesions, conventional ultrasound and other methods can be used as guidance methods, and a 3D-PNCT-guided plate is not necessary); (5) lesion diameter > 1 cm; and (6) expected survival time of more than 3 months. The contraindications were (1) coagulation dysfunction; (2) severe organ function disorder; (3) mental illness; and (4) recent active infection. This study was approved by the Ethics Committee of The First Affiliated Hospital of Zhengzhou University (approval no. 2021-KY-1105-002) and conducted per the Declaration of Helsinki. All patients signed informed consent to participate in this study.

**Table 1:** Characteristics of the 36 patients.

Items	Number/value	%
Median age (years)	44.5 (27-58)	
Pathology		
Squamous cell cancer	27	75.0
Adenosquamous cell cancer	9	25.0
Tumor size		
≥3 cm	27	75.0
<3 cm	9	25.0
ECOG performance status		
0	6	16.7
1	30	83.3
Previous radiotherapy (Gy)		
60.4	7	19.4
55.9	19	52.8
50.0	2	5.6
44.4	8	22.2

### 2.2 Pre-plan system

Patients were fixed with a vacuum pad and underwent an enhanced computed tomography (CT) (SOMATOM Drive, Siemens Inc., Germany) scan with a 0.625-mm slice thickness before brachytherapy. The position points and lines were marked on the patients’ skin according to the tumor location. The CT images were transferred to a BrachyVision system (Varian Inc., USA). Organs at risk (OARs) and high-risk clinical target volume (HR-CTV) were defined and delineated. Based on the principle of the Paris dosimetry system and its optimization (11–13), the needles were placed parallel, and in any plane perpendicular to the needle, the needles were arranged as equilateral triangles with a 8–10-mm spacing between each needle. If the needle passes through important structures (such as bones, blood vessels, nerves, and organs), the position of the needles should be shifted as a whole or the spacing of the needle path should be fine-tuned (ensure that the needle path is arranged in an equilateral triangle, and the spacing is still within 8–10 mm). If the important structure cannot be avoided, the corresponding needle will be discarded. Each needle was first reconstructed using HR-CTV as the reference target. Then, the inverse planning simulated annealing algorithm was selected, and the corresponding parameters such as the minimum dose on the target surface and the maximum dose on the OAR surface were set at the corresponding interface. Finally, the system optimization was performed to obtain the plan. The maximum dose limit of HR-CTV was increased to further meet the clinical requirements, the dose of HR-CTV was further increased without increasing the dose of OAR, and the dose hot and cold points were further reduced. Finally, manual dose line dragging was performed for small adjustment when necessary. The prescribed dose was 600 cGy, and 95% of the target volume was required to receive the prescribed dose. The dose received by the D2cm^3^ of OARs was minimized.

### 2.3 Design and printing of templates

**Figure 1:**
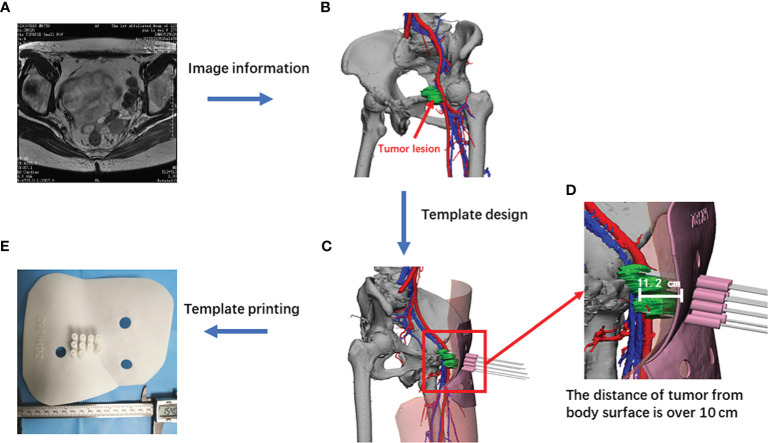
Flowchart of the printing of individualized non-coplanar templates. **(A)** Representative MRI image of non-central recurrent cervical cancer; **(B)** recurrent lesion; **(C)** simulated diagram of template designed with Mimics software (Version 23.0, Belgian); **(D)** enlarged view of tumor lesion; **(E)** a real template printed out.

The image data were imported into Mimics software (Version 23.0, Belgium) to extract information on bones, blood vessels, bladder, rectum, ileum, colon, skin, and tumor lesions. Mimics software measured the number of needles, direction and deviation of needle entrance, and depth and angle in the pre-plan. According to the characteristics of the body surface near the puncture site, the panel was designed to fit closely with the skin, and the positioning markers pre-placed before the CT scan were included for accurate reduction during treatment. A biocompatible nylon material (PA12, Huashu Hi-Tech Corporation in Hunan, China) was selected; the selective laser sintering process was used for printing on a Nylon 3D printer (Huashu Hi-Tech Corporation in Hunan, China), and the printing accuracy was ±0.1 mm (**Figure 1**). The same patient used the same template for each treatment, but each treatment was performed according to the redesigned preplanned data.

### 2.4 Operation process of 3D-PNCT-assisted ISBT

**Figure 2:**
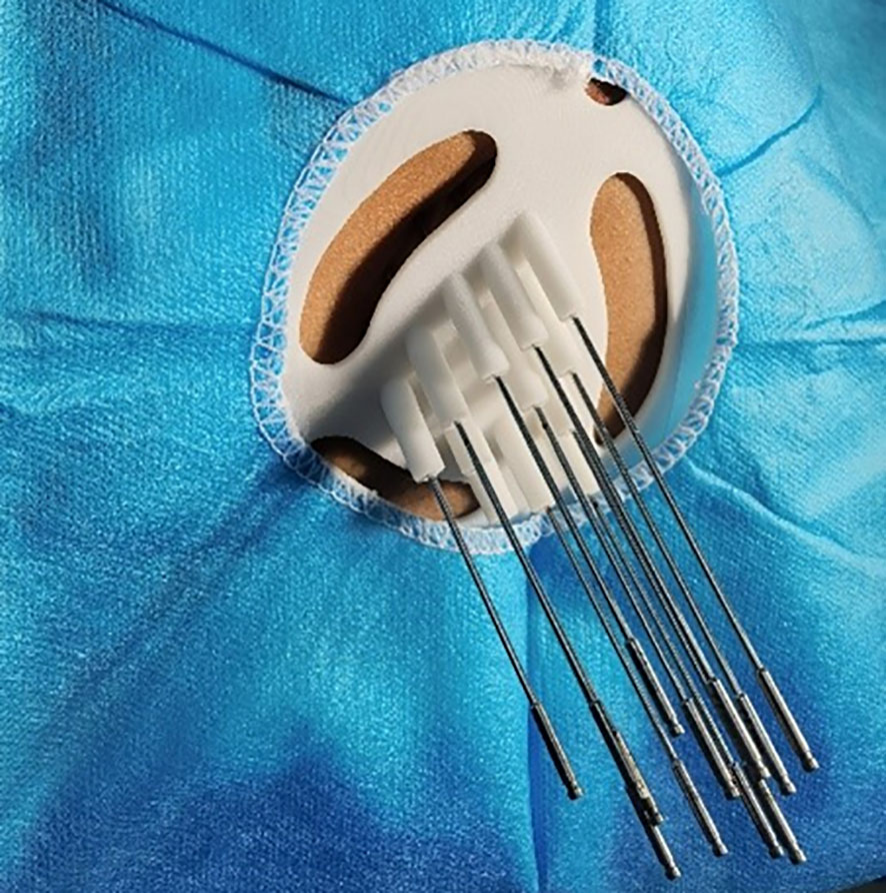
A 3D-printing non-coplanar template was fixed on the patient’s body according to the laser lines and position lines, and the needles were inserted accordingly.

The patient was placed in a prone position, fixed with a vacuum pad, and reset according to the body surface marking line. The patient was then subjected to intravenous general anesthesia. After cleansing the skin well around the venipuncture site and draping the area, the template was placed according to the body surface location. Soon afterward, the needles were inserted individually along the predesigned needle track and needle insertion depth (**Figure 2**).

CT (SOMATOM Drive, Siemens Inc., Germany) with 3.0-mm slice thickness was used to obtain positioning images and confirm good fit of the guide plate to the skin. The needle did not bend and reached the intended position (the needle penetrated the tumor without passing through vital structures). The patient’s vital signs were stable and had no special discomfort symptoms. Then, the images were transmitted to the target-area delineation system. The doctor then outlined the target area and organs at risk. Target volume delineation and planning was performed. Following the consensus of experts, HR-CTV was defined as the range of recurrent lesions, which had the following characteristics: located deep in the abdomen and pelvis and eccentric and adjacent to vital vessels, nerves, and organs. These lesions were usually 10–20 cm from the body’s surface. The surrounding OARs, including the bladder, rectum, ileum, and colon, were also delineated. The bladder included the entire outer wall of the bladder, the lower boundary was located at the beginning of the urethra, the rectum consisted of the outer wall of the rectum, and the lower boundary started from 1 cm above the anus. The upper boundary reached the rectosigmoid junction. When the ileum and colon are adjacent to the recurrent lesions, the lower and upper boundaries ended at the disappearance of the recurrent lesions. The prescribed dose which was mainly determined by the size and depth of the recurrence and its distance from the surrounding vital organs was 6 Gy/time, once a week, five to six times in total. HR-CTV D90 represents the lowest absorbed dose of 90% HR-CTV volume. D2cm^3^ represents the absorbed dose received by 2 cm^3^ of OARs, which was combined with the equivalent dose in 2 Gy per fraction (EQD2) under the total dose of external irradiation and close range. It required rectal D2cm^3^ ≤65–75 Gy, colon and ileum D2cm^3^ ≤70–75 Gy, and bladder D2cm^3^ ≤80–90 Gy.

Next, the physicians designed the treatment plan. In the Oncentra brachytherapy treatment planning system, the detailed description of the treatment planning process was the same as described in the pre-plan.

**Figure 3:**
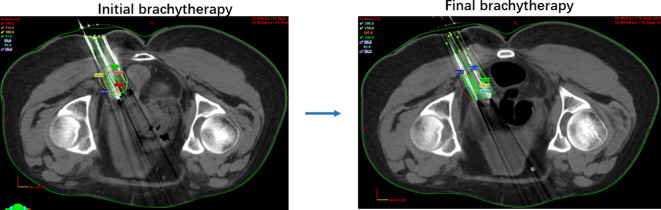
Graphical representations of brachytherapy with PNCT. Preoperation and postoperation validations; the different color lines represent corresponding isodose. Pink, dose 80%; red, 100%; orange, 150%; green, 200%.

Finally, the doctor reviewed the plan and initiated treatment. The make of the high-dose-rate ISBT unit is Varian Oncology (California, USA), and the model is GammaMedplus iX. The radioactive source of GammaMedplus iX is ^192^Ir encapsulated in stainless steel. The diameter of the ^192^Ir pellet for is 0.6 mm, and the active length is 3.5 mm. After treatment, the needles and 3D-PNCT were removed. The gauze compressed and fixed the puncture points. The patient returned to the ward after 20 min of rest in the anesthesia recovery room (**Figure 3**).

### 2.5 Dosimetric and safety evaluation and follow-ups

Dosimetric parameters included HR-CTV D90, target volume (Vt), target volume percentage receiving 100% prescription dose (V100%), target volume receiving 150% prescription dose (V150%), total volume contained in prescription dose (Vref), target volume receiving 100% prescription dose (Vt,ref), target volume receiving 150% prescription dose (Vt,1.5ref), homogeneity index (HI), conformal index (CI), and external index (EI), HI = (Vt, ref-Vt,1.5ref)/Vt,ref * 100%, CI = (Vt,ref/Vt) * (Vt,ref/Vref), EI = (Vref-Vt,ref)/Vt) * 100% (14, 15). D2cm^3^ of the rectum, bladder, ileum, and colon were compared between pre- and posttreatment plans. Data analysis was performed with SPSS (version 26.0, USA), and a p-value < 0.05 was considered significant.

According to the European Organization Research and Treatment of Cancer (EORTC) and Radiation Therapy Oncology Group (RTOG) criteria, perioperative adverse events and acute radiotherapy side effects were evaluated during brachytherapy and within 3 months after brachytherapy.

### 2.6 Statistical analysis

Data analysis was performed with SPSS (version 26.0, USA), and a p-value < 0.05 was considered significant. Categorical variables were described as percentages. Continuous variables were tested for K-S normality. Variables with normal distribution were described by mean ± standard deviation, and those with skewed distribution were described by median and quartiles. The mean was compared by paired sample t-test. Parameters with skewed distribution were tested by Wilcoxon rank-sum test.

## 3 Results

### 3.1 Patient characteristics

The mean age of the patients was 47.11 ± 8.68 years old, all the patients had previously been treated with external radiotherapy, and the median previous radiotherapy dose was 55.90 (50.00–55.90) Gy. The mean time between local recurrence and the end of the last radiotherapy was 15.31 ± 4.25 months, as is shown in **Table 1**. Before enrollment, 31 patients had received second-line or beyond chemotherapy, and 5 patients had received first line chemotherapy. A total of 36 personalized 3D-PNCT guides were fabricated, and all 36 patients underwent successful ISBT under general anesthesia.

### 3.2 Dosimetric parameter analysis

The number of needles per patient was 9.64 ± 2.69. The target volume was 39.77 (28.54–93.36) cm^3^. In terms of tumor irradiated volume, the preoperative and postoperative values of HR-CTV D90, V_100%_, and V_150%_ were (616.97 ± 8.76) cGy and (616.13 ± 8.84) cGy, (*p* = 0.568), 92.53% (91.43%–95.17%) and 92.27% (90.86%–94.48%) (*p* = 0.208), (59.42 ± 4.66)% and (61.34 ± 3.67)% (*p* = 0.002), respectively.

In terms of the parameters of uniformity and accuracy of radiation dose distribution, the preoperative and postoperative values of CI, HI, and EI were 0.72 ± 0.08 and 0.70 ± 0.08 (*p* = 0.345), (35.35 ± 7.75)% and (33.72 ± 4.17)% (*p* = 0.116), (30.60 ± 13.49)% and (28.82 ± 12.71)%, (*p* = 0.471), respectively.

In terms of exposure to OARs, the preoperative and postoperative of D2cm^3^ of the bladder, rectum, ileum, and colon was 110.08(57.51–132.24) and 113.87(65.59–136.95) cGy (*p* = 0.520), 276.51(179.93–335.97) and 266.91(185.85–336.90) cGy (*p* = 0.888), 76.62 ± 46.98 and 77.18 ± 45.66 cGy (*p* = 0.784), 172.04 ± 108.66, and 172.40 ± 110.12 cGy (*p* = 0.859), respectively, as is shown in **Table 2**.

**Table 2:** Comparisons of dosimetric parameters of 3D-PNCT-assist brachytherapy between pre- and postoperation.

Items	Preoperation	Postoperation	*p*
HRCTVD90 (cGy)	616.97 ± 8.76	616.13 ± 8.84	0.568
V_100%_ (%)^#^	92.53 (91.43–95.17)	92.27 (90.86–94.48)	0.208
V_150%_ (%)^##^	59.42 ± 4.66	61.34 ± 3.67	0.002
HI (%)^##^	35.35 ± 7.75	33.72 ± 4.17	0.116
CI^##^	0.72 ± 0.08	0.70 ± 0.08	0.345
EI (%)^##^	30.60 ± 13.49	28.82 ± 12.71	0.471
D2cm^3^ of OARs			
Bladder (cGy)^#^	110.08 (57.51–132.24)	113.87 (65.59–136.95)	0.520
Rectum (cGy)^#^	276.51 (179.93–335.97)	266.91 (185.85–336.90)	0.888
Ileum (cGy)^##^	76.62 ± 46.98	77.18 ± 45.66	0.784
Colon (cGy)^##^	172.04 ± 108.66	172.40 ± 110.12	0.859

^#^, median (interquartile range); ^##^, mean (standard deviation); HRCTV D90, prescribed dose delivered to 90% of the high-risk clinical-target volume; V_100%_ and V_150%_, gross tumor volume receiving 100% and 150% of the prescribed dose respectively; CI, conformation index; EI, external volume index; HI, homogeneity index; OARs, organs at risk.

### 3.3 Safety analysis

There were no serious perioperative complications or grade 3–4 acute radiotherapy side effects. 13.9% of patients had subcutaneous congestion at the puncture site, 8.3% had postoperative pain scores greater than 3 points, 8.3% had grade 1 acute radiation enteritis, 11.1% had grade 2 acute radiation enteritis, and 13.9% had grade 1 acute radiocystitis.

## 4 Discussion

Although ISBT has been used for years to treat cervical cancer, the treatment options for non-centrally recurrent cervical cancer remain limited and unsatisfactory due to the distinctive characteristics of non-central recurrent pelvic lesions (16, 17). These recurrent lesions are 10–20 cm away from the body surface, with vessels, nerves, and important organs around them. The lack of standardized and unified implantation standards makes it difficult to ensure quality control. In addition, the puncture path interferes with OARs and bones, which affects the depth and angle of the needle implantation. In many cases, implantation cannot achieve preoperative planning and design, and the deviation is large.

Moreover, multiple CT scans are required to monitor the position, angle, and depth during the operation, which is inefficient, prolongs the operation time, and increases the risk of complications. Routine implantation on the body surface is difficult for lesions in such a position, and the possibility of trauma is close to 100% (16, 18, 19), making this impossible. These risks can be avoided with the channels on a template. These templates are sculptured to the body’s surface and made with a 3D printer. The 3D printer is a rapid prototyping machine that accumulates layers of materials until the final solid body is made. Currently, 3D bioprinting technology is one of the most popular technologies, and its applications in cell, tissue, organ, and orthopedic implant printing are becoming well established. 3D printing technology has rapidly expanded in the industrial field since its introduction into medicine (20–26).

Before the advent of 3D-printed body surface templates, 3D-printed transvaginal templates were occasionally reported in clinics. Jacob reported that personalized 3D-printed vaginal templates could achieve a reasonable distribution of high doses of radiotherapy for the clinical treatment of cervical cancer (8). Jiang et al. used transvaginal templates during brachytherapy in 32 patients with centrally recurrent cervical cancer (27). They found that three patients (9%) developed grade 3 or 4 late toxicity, and the local remission rate 1 month after the end of treatment was up to 84.4%. The median time to progression (TTP) was 15.4 months (95% CI; 11.3–19.6 months), and the 1-year local control rate (LC) was 51.7%. However, vaginal template implantation is often used to treat superficial recurrent lesions, such as those in the cervix, vagina, and vaginal stump. Still, for non-central pelvic recurrence lesions, the vaginal template is difficult to implant accurately and its efficacy is discouraging (28).

Inspired by previous 3D-printing applications, we applied 3D-printed personalized non-coplanar templates to make a reliable connection between the patient’s anatomical features and the puncture path of the needles into the body; the design parameters were accurately applied to the surgical operation. All the recurrent cases in this study were difficult to treat clinically with expected poor efficacy; all patients had previously received surgery, chemotherapy, or radiotherapy. Therefore, we translated the design parameters into surgical operation, allowing for precise needle implantation during surgery. By designing the surgical template before surgery, we found that the difficulty of surgery was significantly reduced and the operation time was shortened to 10–20 min. Importantly, the patient’s target coverage was improved, allowing the dose received to be closer to the prescription dose. In addition, the low and acceptable adverse effects of the treatment ensured patient compliance, making it possible to treat patients with non-centrally recurrent cervical cancer. Wang et al. used 3D-PNCT-assisted iodine-125 particle implantation to treat recurrent cervical cancer, but they used a permanently implanted radioactive source with a low dose rate (<2 Gy/h), and the radioactive source in the patient’s body would radiate the surrounding environment (10). In this study, we used a similar 3D-PNCT but with a completely different high dose rate (>12 Gy/h) iridium 192 interstitial brachytherapy. The radiation source and needle were removed immediately after treatment. Therefore, in our study, a better effect was expected due to the higher dose of radiation to the tumor, and there was no radiation to the surrounding environment after treatment.

When needles are implanted incorrectly, the postoperative dosimetric parameters may not satisfy the requirements of the initial plan and the outcomes may be compromised. There are three main reasons for the discrepancy between the preplanned and actual exposure doses. First, tumor regression during treatment leads to changes in the position of the implantation needle. Second, intertumoral hemorrhage occurred after the puncture. Third, different degrees of bladder and rectal filling led to alter the location of the tumor target area. Even though we used the same template for each treatment (taking into account the total cost, etc.) for the same patient, each treatment was carried out after reformulating the pre-plan and obtaining the ideal dosimetric parameters in this study.

In this study, there was no statistical difference between the main dosimetric parameters verified after surgery and those before surgery, indicating that the application of templates could realize the relevant parameters of preoperative planning design. The HI data before and after surgery were not ideal, but some studies believed that the high-dose area inside the tumor was more conducive to tumor control to a certain extent when there were no organs at risk within the tumor, suggesting that the clinical significance of HI during ^192^Ir ISBT was not clear, whereas CI and EI were more important evaluation indicators. There was a difference of V_150%_ before and after surgery, indicating that there was still a difference in dose distribution between the actual operation and the preoperative plan. The reason may be that the position error still existed, leading to a slight deviation in the direction or depth of needle insertion. Moreover, due to the influence of tissue density, the needle stress deformation during puncture caused a slight deviation in the direction of implantation path.

It is difficult to determine the optimal prescription dose for re-radiotherapy in patients with non-central recurrent cervical cancer. Mahantshetty et al. observed that the local control rate of treatment dose (EQD2) ≥ 40 Gy was relatively high (52% vs. 34%), and after long-term follow-up, the 2-year local control rate and tumor-free survival rate of patients in this group were significantly improved (29). In order to balance the dose between the target area and the organ at risk, the treatment dose (EQD2) selected in this study was 40–48 Gy and there were no grade 3–4 acute radiotherapy side effects.

There are some limitations to the present study. First, it was a retrospective with a small-sample size, which may have led to potential bias. Second, most patients received brachytherapy combined with systemic therapy, making it difficult to evaluate the efficacy of this therapy on its own. Third, additional research is required to determine whether good dosimetric parameters can prolong the survival of patients.

In summary, with the aid of 3D-PNCT, traditional ISBT can be accurately and safely applied in the treatment of patients with non-central recurrent cervical cancer and can be used as a new local treatment option for this disease.

## Data availability statement

The original contributions presented in the study are included in the article/supplementary material. Further inquiries can be directed to the corresponding authors.

## Ethics statement

The studies involving humans were approved by the Ethics Committee of The First Affiliated Hospital of Zhengzhou University (approval no. 2021-KY-1105-002). The studies were conducted in accordance with the local legislation and institutional requirements. The participants provided their written informed consent to participate in this study. Written informed consent was obtained from the individual(s) for the publication of any potentially identifiable images or data included in this article.

## Author contributions

YC and HS contributed to conception and design. CW and YS contributed statistical analysis and writing of the manuscript. JL, YL and XL contributed data collection.
